# The Effect of Grain Orientation of β-Sn on Copper Pillar Solder Joints during Electromigration

**DOI:** 10.3390/ma15010108

**Published:** 2021-12-24

**Authors:** Kexin Xu, Xing Fu, Xinjie Wang, Zhiwei Fu, Xiaofeng Yang, Si Chen, Yijun Shi, Yun Huang, Hongtao Chen

**Affiliations:** 1 School of Electronics and Information, South China University of Technology, Guangzhou 510640, China; 2 Department of Materials Science and Engineering, Harbin Institute of Technology, Shenzhen 518055, China; 19s155065@stu.hit.edu.cn (K.X.); 19s155068@stu.hit.edu.cn (X.W.); 3 Department of Reliability Design Research, China Science and Technology on Reliability Physics and Application of Electronic Component Laboratory, Guangzhou 510610, China; fuzhiwei@ceprei.com (Z.F.); yxf004@hotmail.com (X.Y.); chensi@ceprei.com (S.C.); shiyijun@ceprei.com (Y.S.); huangyun@ceprei.com (Y.H.); 4 Sauvage Laboratory for Smart Materials, Harbin Institute of Technology (Shenzhen), Shenzhen 518055, China

**Keywords:** copper pillar, electromigration, grain orientation, IMC accumulation, preferred orientation

## Abstract

The grain orientation of Sn-based solder joints on copper pillars under the combined action of electron wind force and temperature gradient greatly affects their electromigration damage. The copper pillars with Sn-1.8Ag lead-free solder on the top was subjected to a current density of 1.5 × 10^4^ A/cm^2^ at 125 °C to study the electromigration behaviors. The grain orientation was characterized by scanning electron microscopy (SEM) equipped with electron backscattered diffraction (EBSD) detector. Metal dissolution and voids formation in the cathode as well as massive intermetallic compounds(IMC) accumulation in the anode were observed after electromigration. Closer examination of solder joints revealed that the Sn grain whose c-axis perpendicular to electric current may have retarded Cu diffusion to anode and IMC accumulation. In addition, the newly formed Cu_6_Sn_5_ exhibited preferred orientation related to the electric current direction.

## 1. Introduction

As the miniaturization and high-performance requirement of microelectronic devices continue to increase, micro-bumps become the mainstream trend in high-density packaging [1,2]. Copper pillar solder joints exhibited excellent properties in electrical conductivity and thermal conductivity, and have been used as the main interconnection method in the case of below 100 μm fine pitch [3,4,5,6,7]. However, the volume of solder used for bonding would shrink significantly, with the transition from C4 joints (controlled collapse chip connection) to copper pillar joints. Many reliability issues emerge owing to the shrinking solder volume [8,9]. One such issue is that the increasing current density through each copper pillar may exceed the electromigration threshold (10^4^ A/cm^2^), which causes the electromigration damage [10].

Most of the lead-free solders used at present are tin-rich, which usually contain more than 90% Sn, indicating that the properties of lead-free solders are dictated by the properties of pure Sn [11,12,13,14]. Due to the downsizing of solder used in copper pillar, there are only limited β-Sn grains. β-Sn has a lattice structure of body-centered tetragonal (a = b = 5.83 Å, c = 3.18 Å), whose physical, chemical properties are highly anisotropic [14,15,16]. When solder joints contain single or several grains, the anisotropy of β-Sn will severely affect the integrity and reliability of lead-free solder joints.

The detailed report of Lu et al. [17] indicated the formation of IMC along grain boundary parallel to the current flow in bi-crystal Sn grain solder joints containing two grains with very different orientations. If the c axis of the grain is more parallel to the current flow, IMC will grow rapidly due to the rapid diffusion of Cu. If the c axis of the grain is more perpendicular to the current flow, IMC accumulation would the suppressed due to the retardation of Cu towards anode. Shen [18] reported that the sample with α angle lower than 25° (α was defined as the angle between c axis of Sn grain and current flow) exhibited fast accumulation of interfacial IMC by drawing a relationship between the average α in the solder joints with the thickness of the interfacial IMC in electromigration experiments on a few solder joints composed of several grains with different α. Huang [19] noticed that when electrons flowed from the β-Sn grains with smaller α to the adjacent grains with high α, a large amount of IMC was accumulated at the grain boundary, because the migration of Cu atoms from the grain boundary to the anode was hindered, and a large amount of columnar Cu_6_Sn_5_ IMCs was precipitated in the β-Sn grains with smaller α. When electrons flowed from the grain with high α to the grain with smaller α, voids at the interface of Cu-solder formed and little IMC grew. Tian et al. [20] found that IMC growth can be seen when the c axis of the Sn grains was aligned with the electron flow direction towards the cross-sectional surface, as they could form a convex bulge on the surface of the sample. Additionally, the IMC growth behavior of one grain was not affected by the other grains in the single solder joint.

Although copper pillars are a popular interconnection method in 3-D packaging, there are few studies on the electromigration damage in this kind of solder joint. However, due to the limited solder volume and anisotropic property of each interconnect joint, chemical reactions may convert all solder to brittle intermetallic compounds. In this study, electromigration tests were conducted with the sample of Cu/Sn-1.8Ag/Cu microbumps under current density of 1.5 × 10^4^ A/cm^2^ at 125 °C. SEM and EBSD images were utilized to reveal the relationship between electromigration damage and tin grain orientation.

## 2. Experiment

**Figure 1:**
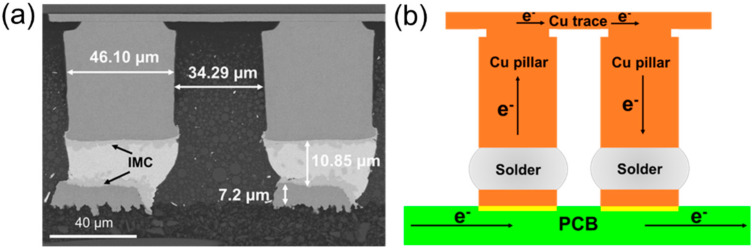
The structure of tested copper pillar joint: (**a**) The dimension and structure of the test samples after reflow; (**b**) the schematic diagram of experiment setup.

Figure 1a showed the dimension and structure of the copper pillar samples. The solder cap was electroplated on the copper pillar, and then the solder joint was reflowed to form a complete joint, which was shown in Figure 1a. The solder for bonding was Sn-1.8Ag solder with height of 23.10 μm before joining. A small amount of IMC was formed at the interface between Cu substrate and solder during the bump fabrication. The diameter of copper pillar was 46.10 μm, which was same as the diameter of solder mask. On the substrate of BT material side, the copper trace was 7.2 μm thick. The pitch between two adjacent pillars was 34.29 μm.

The applied current density loaded on copper pillar was 1.5 × 10^4^ A/cm^2^ at 125 °C, the schematic diagram of experiment setup was shown in Figure 1b. After grinding and polishing, SEM and EBSD were conducted to examine microstructure evolution and the grain orientation, respectively. For easy understanding of Sn grain orientation, α was defined as the angle between the c-axis of β-Sn and electron flow, describing the orientation of Sn grain in this study.

## 3. Results

### 3.1. Microstructure Evolution of Cu Pillar after Electromigration Test

**Figure 2:**
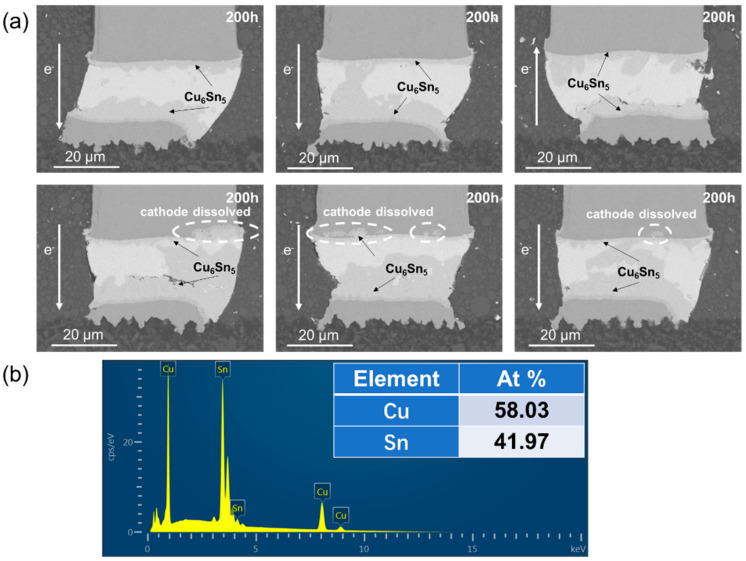
Microstructure after current stressing at 1.5 × 10^4^ A/cm^2^ at 125 °C for 200 h: (**a**) SEM images of copper pillar joints (**b**) the EDS results of IMCs.

During the electromigration, atoms at the cathode diffused to the anode, forced by electron wind under the action of electrothermal coupling. As shown in Figure 2, the Ni layer and the Cu layer at the cathode were dissolved to different extent, dominated by the Ni and Cu flux induced by the current after current stressing at 1.5 × 10^4^ A/cm^2^ at 125 °C for 200 h. Ni and Cu are interstitially diffused in the Sn matrix due to their low solubility in Sn [21,22]. The anode received a great number of Cu atoms from the cathode and a great amount of IMC accumulated, which mainly consisted of Cu_6_Sn_5_ and Cu_3_Sn. Cu_6_Sn_5_ and Cu_3_Sn were both the phases with high hardness and low strength, which could be the potential site for crack initiation. Under the action of stress, crack initiation and propagation may occur in the IMC layer. The fracture mode of solder joint may change from plastic mode to plastic-brittle mixed mode. The accumulation of Cu_6_Sn_5_ and Cu_3_Sn had great influence on the reliability of copper pillar joints.

### 3.2. The Effect of Grain Orientation on Electromigration Damage

**Figure 3:**
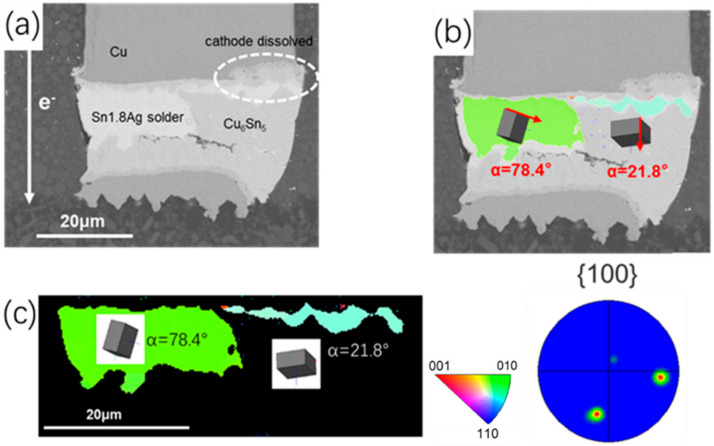
Microstructure after current stressing at 1.5 × 10^4^ A/cm^2^ at 125 °C for 200 h: (**a**) SEM image for the bump with downward electron flow; (**b**) SEM image for the bump with downward electron flow with unit cells; (**c**) the EBSD image corresponding to (**a**).

**Figure 4:**
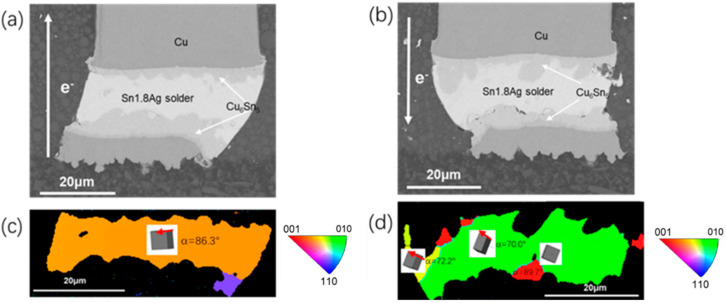
Microstructure after current stressing at 1.5 × 10^4^ A/cm^2^ at 125 °C for 200 h: (**a**) SEM image for the copper pillar with upward current flow; (**b**) SEM image for the copper pillar with downward current flow; (**c**) the EBSD image corresponding to (**a**); (**d**) the EBSD image corresponding to (**b**).

The inherent intrinsic properties of β-Sn were key factors to the interstitial diffusion of Ni, Cu. The diffusion coefficients of Cu and Ni in the c axis could be 43 times and 3 × 10^4^ times than that in other directions at 150 °C, respectively [21,23]. Thus, characterization of grain structure was necessary for study on electromigration. α was defined as the angle between c axis of Sn grain and electric current. As shown in Figure 3c, only two grains with different orientations were observed in the tested Sn matrix. The angle α of the right blue β-Sn grain was 21.8°, massive Cu_6_Sn_5_ IMC almost occupied the whole grain after 200 h electromigration test. In addition, the Cu pillar was partly dissolved in the right corner. The angle α of the green β-Sn grain was 78.4° and the dissolution of the cathode Cu pillar was greatly retarded. Characterization for other interconnects under same test condition indicates that the two solder joints were comprised of grains with large α, shown as Figure 4b,d. The angle α of the yellow β-Sn grain was 86.3°, which mean the c axis of this grain was almost perpendicular to the electron flow direction. For the other solder joint, three differently oriented grains were all with large α (70°, 89.7°, 72.2°). Due to the lower diffusivity of Sn with orientation of large α, the dissolution of the cathode Cu was limited and fewer IMC accumulated at the anode.

**Figure 5:**
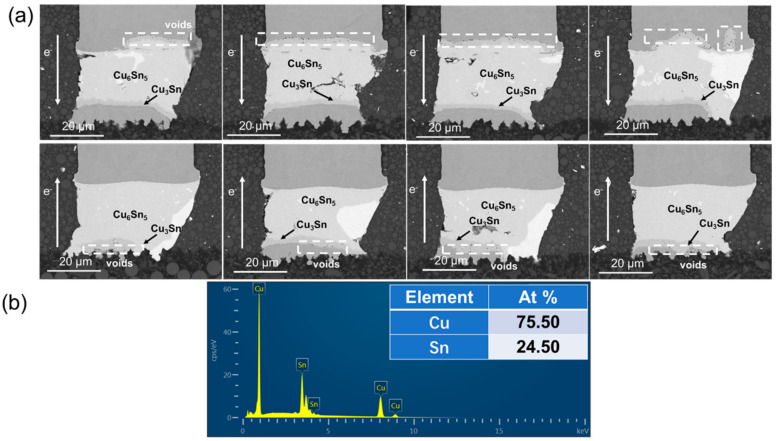
Microstructures of copper pillars full of IMCs after current stressing at 1.5 × 10^4^ A/cm^2^ at 125 °C: (**a**) SEM images of copper pillar joints (**b**) the EDS results of IMCs.

**Figure 6:**
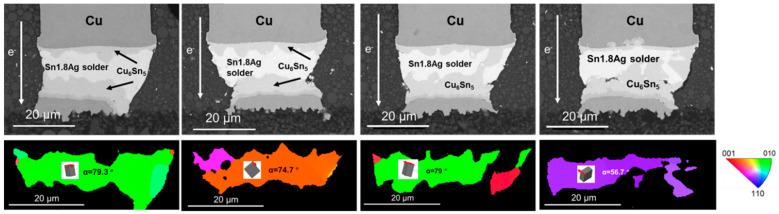
Microstructures of copper pillars with remained Sn after current stressing at 1.5 × 10^4^ A/cm^2^ at 125 °C.

To explore further into the effect of Sn orientation on the migration rate of Cu, the current stressing was terminated till 400 h. The tested sample was electrically stressed under 1 × 10^4^ A/cm^2^ at 150 °C for 400 h. It turned out to that most of solder joints had changed to be full of IMC. From the SEM images of the copper pillar joints in Figure 5, it can be found the solder joints have taken on the structure of Cu/Cu_3_Sn/Cu_6_Sn_5_/Cu_3_Sn/Cu regardless of the current direction in the solder joints. However, under the same condition, many solder joints were still composed of Cu_3_Sn/Cu_6_Sn_5_/Sn/Cu_6_Sn_5_/Cu_3_Sn/Cu. As shown in Figure 6, in the unconsumed Sn, c axis of Sn grain was almost perpendicular to the electron flow. It can be been in the solder joint with the grain orientation of large α, there were many Sn matrix remained due to their stronger electromigration resistance. It was speculated that the reason for the solder joint full of IMC was that c axis of the Sn grain was closer to the current flow. In the cathode, some voids formed, which were caused by the accumulation of vacancies. The copper and Sn atoms were gradually diffused to the anode by the electric current stressing, and in turn, the diffusion of the atoms resulted in a reverse vacancy flux. This flux of vacancies accumulated and formed voids eventually at the cathode.

### 3.3. The Grain Structure of Cu_6_Sn_5_ during Electromigration

**Figure 7:**
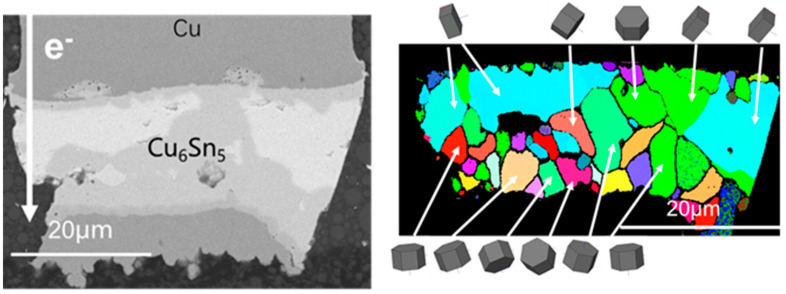
Microstructure after current stressing at 1.5 × 10^4^ A/cm^2^ at 125 °C for 600 h.

The grain structure of Cu_6_Sn_5_ is critical for long-term service because the solder joints were likely to become full of IMC after a long period of operation. Microstructure morphology and grain structure of solder joints tested by electromigration for 600 h under 1 × 10^4^ A/cm^2^ at 150 °C was shown in Figure 7. Several Sn grains whose orientations were unfavorable to the diffusion of copper atoms still existed, but most part of the solder joint had changed into IMC. A small amount of Cu_3_Sn was found at the anode and most of IMC was Cu_6_Sn_5_, which was characterized to show hexagonal grain structure. Additionally, according to EBSD analysis, the newly formed Cu_6_Sn_5_ exhibited preferred orientation related to the current direction. The (0001) plane of Cu_6_Sn_5_, the base of hexagonal grain structure, was considered to be the main plane through which electrons pass [21]. The carrier mobility was the main factor that affects the conductivity because the carrier concentration in a specific metal was close to a constant. In fact, the carrier mobility was inversely proportional to the resistivity, which depended mainly on the scattering of electrons by the lattice. When electrons passed through a crystal plane with the lowest atomic density, they have the least lattice scattering, thus the direction perpendicular to that plane was the lowest resistance path. Therefore, the Sn grain whose (0001) crystal plane perpendicular to the current carries the lowest lattice scattering intensity and the largest diffusion flux of Cu atoms. The grains with lower resistivity would have greater current stress and hence higher atomic flux, so would grow preferentially by swallowing the grains with large resistivity. Therefore, this may be the reason for the preferential orientation of η-Cu_6_Sn_5_ under current stress. However, the grain orientation of Cu_6_Sn_5_ closer to the substrate was rather disordered, which was probably owing to the preferred orientation of Cu_6_Sn_5_ and copper substrate during solder joint reflow. As electric current stressing continued to load, large grains of Cu_6_Sn_5_ grew by swallowing the small ones, finally leading to the disappearance of fine grains. During aging, the newly formed Cu_6_Sn_5_ in the middle exhibited preferred orientation related to the current direction more obviously. Thus, it can be concluded that the grain boundaries of Cu_6_Sn_5_ moved by merging the fine grains with the random orientations.

### 3.4. Microhardness and Elestic Modulus of IMCs in Copper Pillar Joint after Electromigration Test

**Figure 8:**
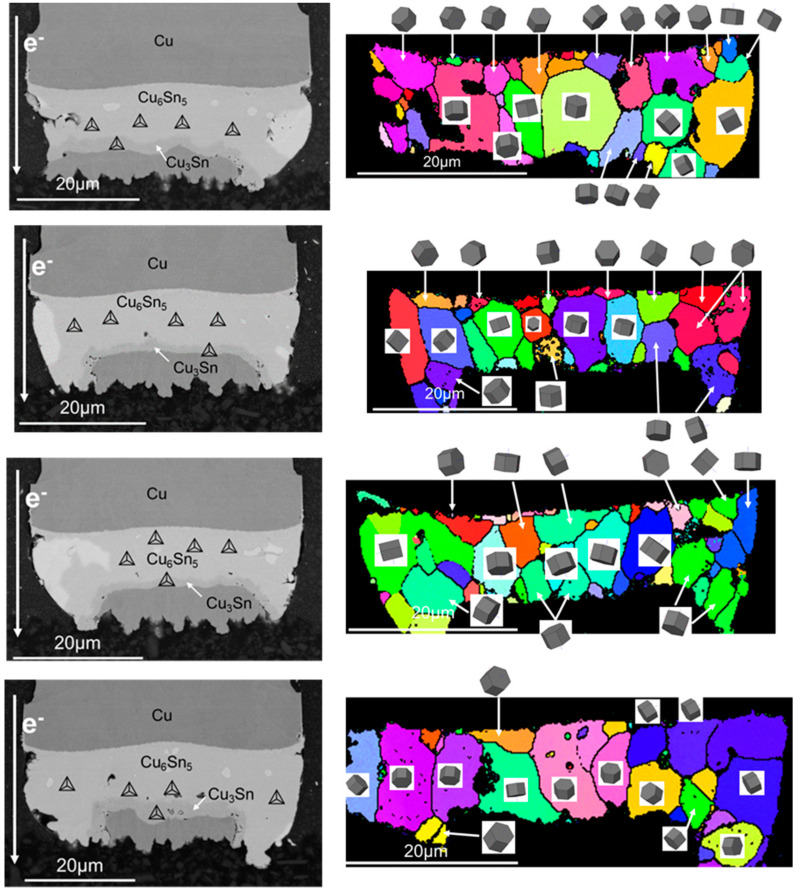
Microstructure after current stressing at 1.5 × 10^4^ A/cm^2^ at 125 °C for 800 h.

**Figure 9:**
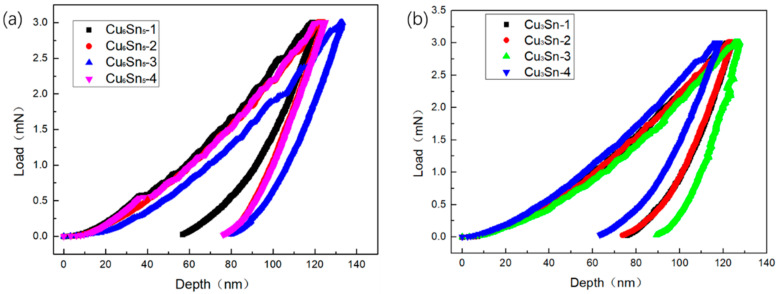
Curves of load vs indentation depth in nanoindentation experiment:(**a**) Cu_6_Sn_5_; (**b**) Cu_3_Sn.

Microstructure morphology, nanoindentation on Cu_6_Sn_5_ and Cu_3_Sn and grain structure of solder joint tested by electromigration for 800 h under 1 × 10^4^ A/cm^2^ at 150 °C was shown in Figure 8. The curve describing relationship between the value of load and indentation depth applied in nanoindentation experiment was shown in Figure 9. Sixteen different positions on Cu_6_Sn_5_ from four different solder joints was selected for indentation experiments to obtain Young’s modulus and microhardness of Cu_6_Sn_5_. According to the Oliver–Pharr method, the hardness, H, was expressed as [24]

$$H=\frac{P_{max}}{A\left(h_{c}\right)}$$

where *A(h_c_)* was the contact area and *P_max_* was the maximum load. To obtain the Young’s modulus *E*, *Er* was indispensable to know, defined as the reduced Young’s modulus by elastic recovery of the sample and the indentation, which can be calculated by [24]

$$E_{r}=\frac{\sqrt{\pi }}{2\beta }\frac{S}{\sqrt{A}}$$

where *β* was a contant relevant to the indentation, *S* was the slope of the top point of the unloading curves of Load–Depth, and *A* was the project contact area. Thus, the Young’s modulus *E* can be obtained by [24,25]

$$E_{r}={\left(\frac{E}{1-\nu^{2}}+\frac{E_{I}}{1-\nu_{I}^{2}}\right)}^{-1}$$

where the and *ν* and *ν_I_* were Poisson’s ratio of sample and the indentation. E_I_ was the Young’s modulus of the indentation. The results showed the average values of Young’s modulus and microhardness of Cu_6_Sn_5_ were 118.24 ± 6.42 GPa and 6.72 ± 0.54 GPa, respectively. The same experiment was also conducted on Cu_3_Sn. Ten different positions on Cu_3_Sn from four different solder joints was selected for indentation experiments to measure Young’s modulus and microhardness of Cu_3_Sn. The results showed the average values of Young’s modulus and microhardness of Cu_6_Sn_5_ were determined to be 147.67 ± 6.42 GPa and 6.46 ± 0.54 GPa, respectively. According to the reports of Deng et al. [26] the Young’s modulus and microhardness of Cu of the pillar was 116.50 ± 4.70 GPa and 1.65 ± 0.17 GPa. The result of nanoindentation experiment revealed that there is Young’s modulus mismatch between Cu_6_Sn_5_, Cu_3_Sn and Cu, which provided additional degradation mechanism.

## 4. Conclusions

The microstructure and grain evolution of copper pillar solder joints after current stressing at 1.5 × 10^4^ A/cm^2^ at 125 °C was studied by SEM and EBSD. During the electromigration, the anode received a large number of Cu atoms from the cathode and a large amount of IMC accumulated, which mainly consisted of Cu_6_Sn_5_ and Cu_3_Sn. Kirkendall voids at the cathode formed due to the vacancies flux. The orientation of Sn grain had a considerable effect on diffusion of Cu and electromigration damage. The Sn grain with the large α effectively retarded the Cu diffusion and IMC accumulation, exhibiting excellent electromigration resistance performance. EBSD analysis showed that the preferred orientation of hexagonal Cu_6_Sn_5_ after electromigration was (1000). The nanoindentation experiment results of newly formed IMC, Cu_6_Sn_5_ and Cu_3_Sn, revealed Young’s modulus mismatch existed between Cu_6_Sn_5_, Cu_3_Sn and Cu, which provided additional degradation mechanism. This study provided better understanding of the microstructure/grain structure change and its effect on IMC formation in solder joint during electromigration.
